# *Wolbachia* and host germline components compete for kinesin-mediated transport to the posterior pole of the *Drosophila* oocyte

**DOI:** 10.1371/journal.ppat.1007216

**Published:** 2018-08-15

**Authors:** Shelbi L. Russell, Nassim Lemseffer, William T. Sullivan

**Affiliations:** Department of Molecular Cell and Developmental Biology, University of California Santa Cruz, Santa Cruz, California, United States of America; Pennsylvania State University, UNITED STATES

## Abstract

Widespread success of the intracellular bacterium *Wolbachia* across insects and nematodes is due to efficient vertical transmission and reproductive manipulations. Many strains, including wMel from *Drosophila melanogaster*, exhibit a specific concentration to the germplasm at the posterior pole of the mature oocyte, thereby ensuring high fidelity of parent-offspring transmission. Transport of *Wolbachia* to the pole relies on microtubules and the plus-end directed motor kinesin heavy chain (KHC). However, the mechanisms mediating *Wolbachia’s* association with KHC remain unknown. Here we show that reduced levels of the host canonical linker protein KLC results in dramatically increased levels of *Wolbachia* at the oocyte’s posterior, suggesting that KLC and some key associated host cargos outcompete *Wolbachia* for association with a limited amount of KHC motor proteins. Consistent with this interpretation, over-expression of KHC causes similarly increased levels of posteriorly localized *Wolbachia*. However, excess KHC has no effect on levels of Vasa, a germplasm component that also requires KHC for posterior localization. Thus, *Wolbachia* transport is uniquely KHC-limited because these bacteria are likely outcompeted for binding to KHC by some host cargo/linker complexes. These results reveal a novel host-symbiont interaction that underscores the precise regulation required for an intracellular bacterium to co-opt, but not disrupt, vital host processes.

## Author summary

The intracellular bacterial symbiont *Wolbachia* uses host motor proteins for microtubule-based transport to the posterior pole of the developing host oocyte, coincident with the future germline, and yet it does not interfere in this process. We present evidence here that *Wolbachia* competes poorly with key host cargos for access to one of these motor proteins, making *Wolbachia* transport limited by its availability. Given that intracellular pathogens tend to be effective competitors for their host proteins, these results suggest that the vertically transmitted bacterium *Wolbachia* has evolved as a weak competitor, potentially to mitigate its impact on normal host biology, and thus increase its own reproductive success.

## Introduction

The intracellular bacterium *Wolbachia* is a widespread vertically transmitted endosymbiont present in the majority of insect and filarial nematode species. In many of these associations, *Wolbachia* appears to confer little benefit to its host, while often incurring large costs [1,2]. Given that *Wolbachia* requires the host for reproduction, yet generally provides little incentive for the host to maintain it, the bacterium has evolved ways of ensuring its transmission through host populations [2].

*Wolbachia* is found in the germline stem cells of *Drosophila* ovaries and exhibits coordinated movements at specific developmental stages [3]. Early events are mediated by the microtubule minus-end directed motor dynein [4] and later events by the plus-end directed motor kinesin [5]. Starting in late stage 9, the wMel strain uses kinesin heavy chain (KHC) proteins for transport to the posterior pole coincident with the assembling germplasm. This localization presumably confers efficient vertical transmission, as *Wolbachia* in this region become incorporated in the germline of the next generation [5]. Significantly, key components of the germplasm also rely on KHC for transport and concentration at the posterior pole [6].

The mechanisms used by *Wolbachia* to associate with KHC are unknown. Although KHC can bind cargo directly [7], the linker protein, kinesin light chain (KLC) is thought to be necessary for much of KHC transport [8,9]. Previous studies of intracellular pathogens revealed evidence for association with both KLC [10] and KHC [11]. Thus, both mimicry of and direct binding to host linker proteins, such as KLC, are possible strategies for an intracellular bacterium to interact with host KHC proteins.

The concentration of *Wolbachia* in the newly formed germplasm of the *Drosophila* oocyte enabled us to explore how endosymbionts engage host processes and integrate into core structures without disrupting function. In fact, *Wolbachia* concentrations must reach extremely high levels before disrupting development [12]. Here we investigate the basis of *Wolbachia’s* association with KHC in the developing oocyte of *D*. *melanogaster*, and reveal a novel molecular competitive interaction between host and symbiont. We provide evidence that *Wolbachia* achieves its normal posterior concentration by being a weak competitor for KHC and its linker proteins, thus ensuring that poleward transport of essential host germline components is not disrupted.

## Results

### Quantification of *Wolbachia* abundance and distribution in oocytes

Unless otherwise indicated, the studies presented here focused on stage 10a of *Drosophila* oogenesis because this time point is well-defined, occurs after the beginning of *Wolbachia* localization at stage 9 [5], and is before fast cytoplasmic streaming begins in stage 10b [13]. We quantified *Wolbachia* using fluorescence intensity of propidium iodide (PI) stained oocytes, as previously described [5] (S1A–S1C Fig). *Wolbachia* were quantified in the whole oocyte, the oocyte posterior, and the posterior pole, adjacent to the cortex (S1D and S1E Fig).

**Fig 1:**
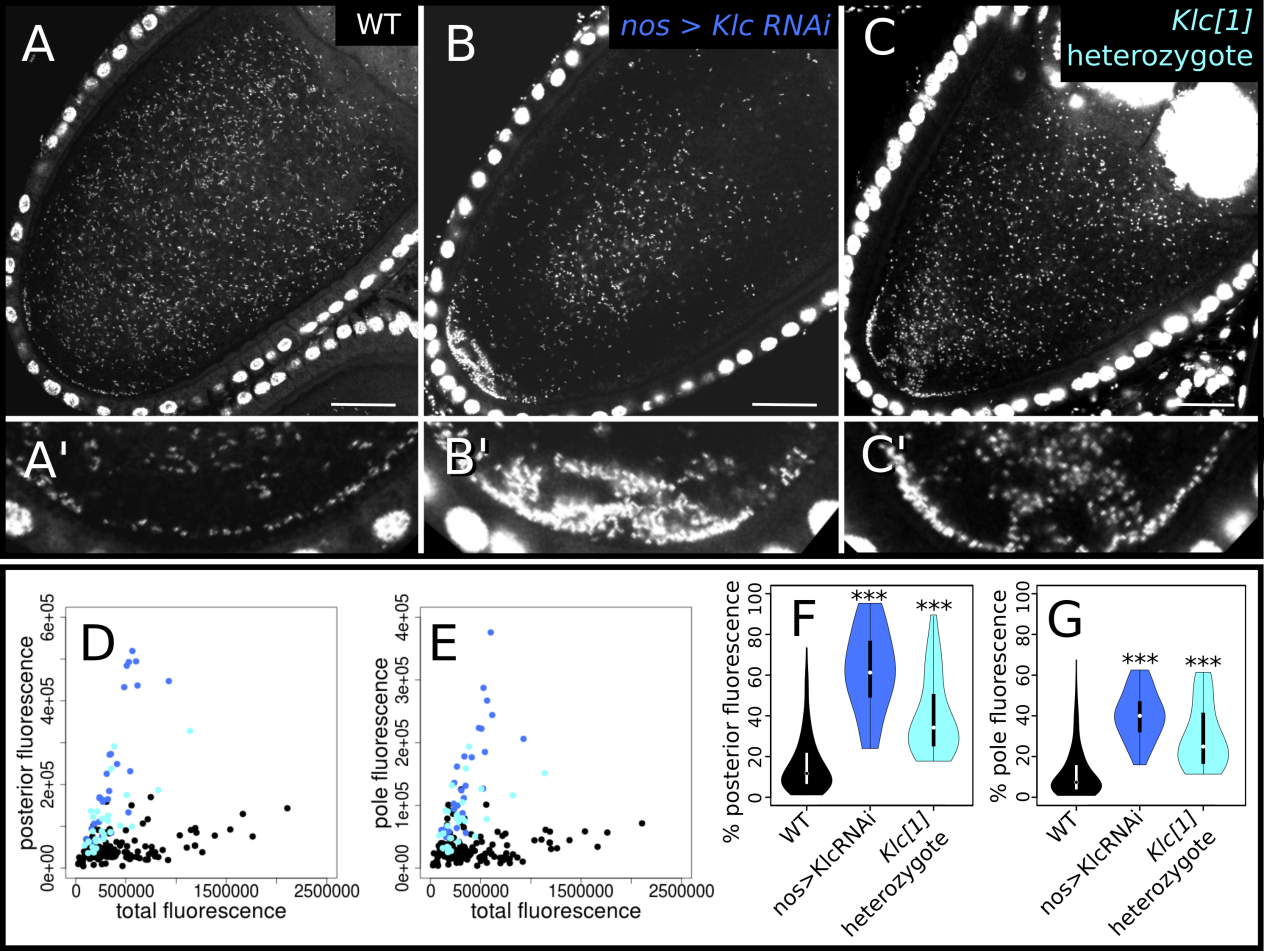
Knockdown of kinesin light chain (KLC) increases wMel *Wolbachia* abundance at the posterior pole in stage 10a oocytes. (A-C) Confocal micrographs of *D*. *melanogaster* oocytes stained with propidium iodide (PI) showing representative examples of (A) Wild-type (WT) localization of *Wolbachia*, and (B,C) increased posterior abundance of *Wolbachia* when KLC is knocked down with (B) RNAi driven by the nanos promoter and (C) a heterozygous null mutation. (A’-C’) Magnified views of pole region in A-C, respectively. (D-G) Quantification of PI fluorescence due to *Wolbachia*. Plots are colored according to their genotype label colors in A-C. (D,E) Total fluorescence was plotted against the amount of fluorescence localized to the (D) oocyte posterior and (E) posterior pole. (F,G) Violin plots of the relative proportion of Wolbachia at the (F) posterior and (G) posterior pole in each of the genotypes listed on the x-axes. Genotypes that contained significantly different posterior abundances than WT in Wilcoxon rank sum tests: *** p < = 0.0001. Scale bars = 25 μm.

**Fig 2:**
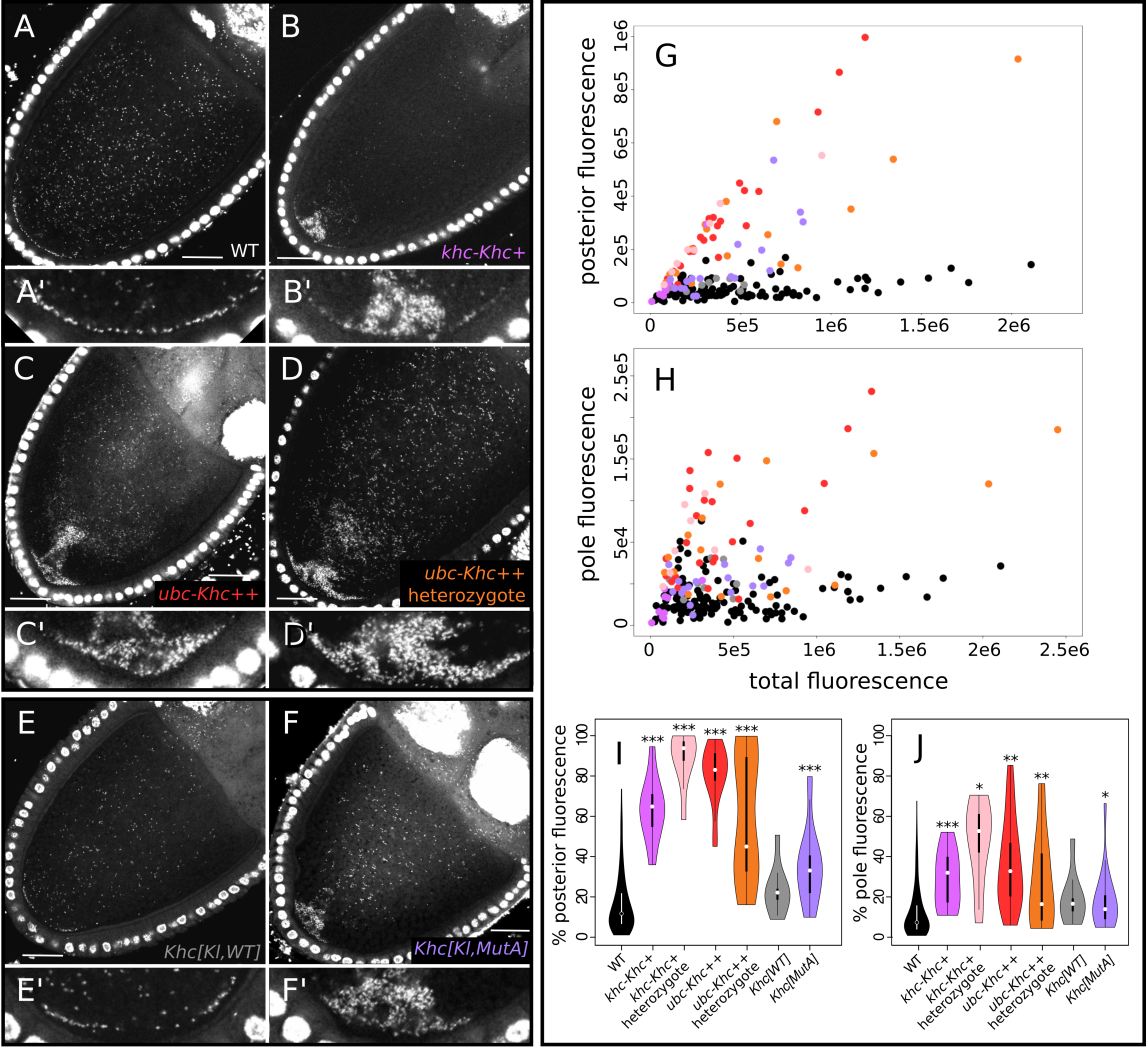
Overexpression of kinesin heavy chain (KHC) and loss of KHC-mediated microtubule sliding increases wMel Wolbachia abundance at the posterior pole in 10a oocytes. (A-F) Confocal micrographs of *D*. *melanogaster* oocytes stained with propidium iodide (PI) showing representative examples of (A) Wild-type (WT) localization of *Wolbachia*, and (B-D) increased posterior abundance of *Wolbachia* when KHC is overexpressed with (B) the native KHC promoter and (C, D) the ubiquitin (*ubc*) promoter, both in (C) homozygotes and (D) heterozygotes. (E) Wild-type localization of Wolbachia in in flies expressing a control transgenic construct containing an inserted wild-type KHC allele. (F) Excess posterior localization of *Wolbachia* in transgenic oocytes containing KHC lacking the microtubule-binding domain (see Fig 5B). (A’-F’) Magnified views of pole region in A-F, respectively. (G-J) Quantification of PI fluorescence due to *Wolbachia*. Plots are colored according to their genotype label colors in A-F. (G,H) Total fluorescence was plotted against the amount of fluorescence localized to the (G) oocyte posterior and (H) posterior pole. (I,J) Violin plots of the relative quantification of the proportion of *Wolbachia* at the (I) posterior and (J) posterior pole in each of the genotypes listed on the x-axes. Genotypes that contained significantly different posterior abundances than WT in Wilcoxon rank sum tests: * p < = 0.01; ** p < = 0.001; *** p < = 0.0001. Scale bars = 25 μm.

**Fig 3:**
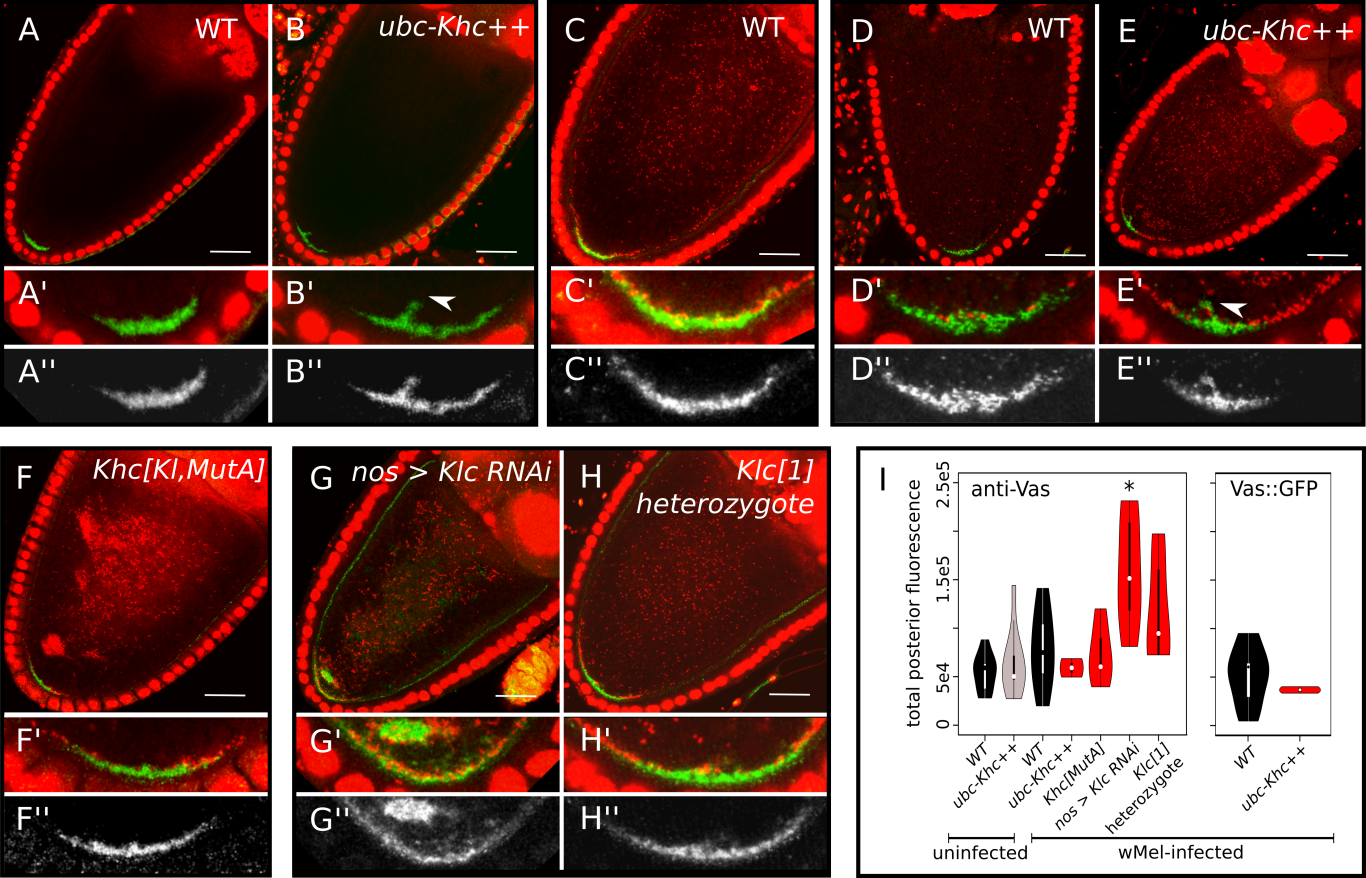
Overexpression of kinesin heavy chain (KHC) does not significantly increase localization of the pole plasm component Vasa (Vas) in stage 10a oocytes. (A-H) Confocal micrographs of fixed *D*. *melanogaster* oocytes with Vasa protein localized by (A-C and F-H) immunolabeling (anti-Vas) or (D,E) Vas::GFP transgene, and stained with propidium iodide (PI, red). Except for (A,B), which are uninfected, all other oocytes are infected with wMel *Wolbachia*. Magnified views of (A’-H’) pole region in (A-H) and (A”-H”) pole region in for Vasa-labelling only, respectively. Arrowheads mark pole plasm drifting off of posterior pole. (I) Quantification of Vas fluorescence signal at the oocyte posterior pole in A-H and S3G and S3H Fig. Violin plots are color-coded for wild-type oocytes (black) and test genotypes with (red) and without (grey) *Wolbachia*. Genotypes that contained significantly different posterior abundances than WT in Wilcoxon rank sum tests: * p < = 0.01. Scale bars = 25 μm. See also S2 Table.

**Fig 4:**
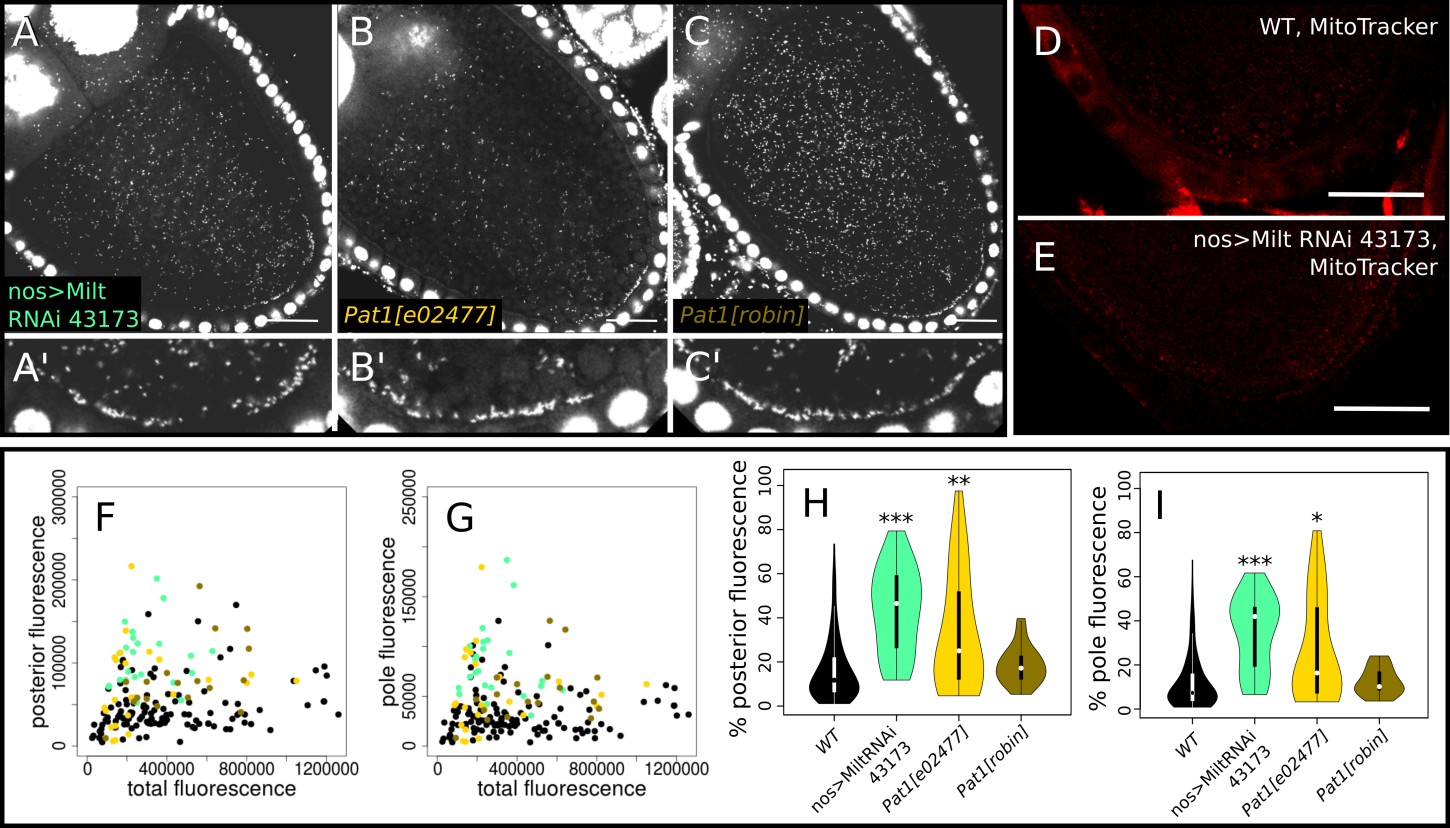
Knockdown of Milton produces a slight increase in wMel *Wolbachia* abundance at the posterior pole in stage 10a oocytes, whereas knockdown of Pat1 does not produce a consistent effect. (A-C) Confocal micrographs of *D*. *melanogaster* oocytes stained with propidium iodide (PI) showing representative examples of increased posterior abundance of *Wolbachia* when (A) Milton is knocked down with RNAi (Bloomington stock 43173) driven by the *nanos* promoter and Pat1 is knockdown with (B) an insertion allele, or (C) a null mutation. (D,E) Live MitoTracker Red staining of (D) WT and (E) Milton RNAi stage 10a oocytes, showing less mitochondrial staining at the posterior pole when Milton is knocked down, indicating the RNAi construct is functional. (F-I) Quantification of PI fluorescence due to *Wolbachia*. Plots are colored according to their genotype label colors in A-C, and wild-type is in black. (F,G) Total fluorescence was plotted against the amount of fluorescence localized to the (F) oocyte posterior and (G) posterior pole. (H,I) Violin plots of the relative proportion of *Wolbachia* at the (H) posterior and (I) posterior pole in each of the genotypes listed on the x-axes. Genotypes that contained significantly different posterior abundances than WT in Wilcoxon rank sum tests: * p < = 0.01, ** p < = 0.001, *** p < = 0.0001. Scale bars = 25 μm.

In wild-type oocytes 16.4 +/- 14.4% of *Wolbachia* resided at the oocyte posterior and 12.3 +/- 12.6% at the pole (n = 125). The small differential between these numbers, 4.1%, reflects that the majority of fluorescence in the posterior region is associated with the pole cortex, with few fluorescent puncta in the space immediately anterior to the pole. See S1 Fig for method of scoring posterior-localized and cortex-associated *Wolbachia*. Throughout the rest of the oocyte cytoplasm *Wolbachia* were evenly distributed, approximately one to two microns apart. These wild-type data were aggregated from the wild-type controls run alongside each genotype dissection, fixation, staining, and imaging run, and are presented in the plots in Figs 1–4 and S4 Fig. Values for total, posterior, and pole fluorescence are presented in S1 Table.

### Knockdown of KLC increases *Wolbachia* posterior abundance

We tested the effect of KLC on *Wolbachia* transport, as it is involved in much of KHC-dependent transport in the host [14]. Unexpectedly, we found that the average oocyte posterior contained 62.4 +/- 20.5% and the pole contained 40.3 +/- 12.3% of total fluorescence in KLC RNAi knockdowns. These concentrations of *Wolbachia* are both significantly more than seen in wild-type oocytes (16.4 +/- 14.4% and 12.3 +/- 12.6% respectively) (n = 26, p < = 1.70E-13 and 3.48E-12, respectively, Fig 1A, 1B and 1D–1G). Furthermore, this pattern is dosage-sensitive, as heterozygotes for the null allele *Klc[1]* exhibited an intermediate phenotype (Fig 1C and 1D–1G), with 40.0 +/- 19.4% of total *Wolbachia* fluorescence at the posterior and 30.1 +/- 15.8% at the pole, both significantly greater than wild-type (n = 27, p < = 6.91E-10 and 7.36E-09, respectively).

Importantly, the enrichment in *Wolbachia* localization was far more pronounced in the region near the oocyte posterior pole (Fig 1D and 1F) than at the pole itself (Fig 1E and 1G), suggesting that this pattern is mediated by transport-specific processes rather than binding processes at the cortex. Total *Wolbachia* abundance in the oocyte was also not significantly different from wild-type in any of the KLC knockdown genotypes (n = 26 and 27, p < = 0.963 and 0.0722 for RNAi and *Klc[1]*, respectively). Furthermore, *Wolbachia* abundance in *Klc RNAi* nurse cells was also not significantly different from wild-type (n = 6, p < = 0.494; S2 Fig). One explanation for these observations is that *Wolbachia* competes with host cargo/KLC linker complexes in association with KHC and for subsequent KHC-driven transport to the oocyte posterior. Thus, reduction of KLC increases the effective amount of KHC available to *Wolbachia* for poleward transport.

### Overexpression of KHC also increases *Wolbachia* posterior abundance

To ascertain whether *Wolbachia* transport is limited by KHC availability, we assayed *Wolbachia* localization when KHC is overexpressed using two transgenic *Drosophila* KHC overexpression constructs [15,16]. Fig 2A–2D depict and G-J quantify *Wolbachia* posterior localization in wild-type oocytes and oocytes with excess KHC. Quantification of posteriorly localized *Wolbachia* in KHC over-expression with the native promoter yields 63.6 +/- 15.9% at the posterior and 28.9 +/- 14.4% at the pole compared to wild-type levels of 16.4 +/- 14.4% and 12.3 +/- 12.6% respectively (Fig 2B). In addition, oocytes derived from females homozygous (C) or heterozygous (D) for KHC overexpression driven by the ubiquitin promoter yield 80.6 +/- 14.7% and 60.6 +/- 31.1% of *Wolbachia* at the posterior, respectively, and 36.7 +/- 21.8% and 27.6 +/- 23.5% at the pole, respectively. All of these values are significantly greater than wild-type (Wilcoxon Rank Sum p << 0.0001 for posterior % and p <<0.01 for pole %; see S1 Table). As with KLC knockdown, transport to the posterior region is more significantly increased than transport to the posterior pole cortex (Fig 2I and 2J).

Interestingly, in three of six stage 10b or older *ubc-Khc++* oocytes examined, excess posterior *Wolbachia* appear to drift away from the posterior pole in aggregate (see S3A and S3B Fig), presumably due to the onset of fast cytoplasmic streaming. This process may ameliorate the effects of excess transport, as embryonic pole cells appeared to contain equivalent amounts of *Wolbachia* in wild-type and KHC overexpression backgrounds (S3C–S3F Fig), and neither *Wolbachia* transmission or host fecundity appear affected in a homozygous *ubc-Khc++* overexpression stock after nine-months of infection (personal observation).

### Elimination of KHC-microtubule binding increases *Wolbachia* posterior localization

In addition to direct transport, *Drosophila* oocytes use KHC for cytoplasmic streaming, which distributes cytoplasmic components throughout the oocyte in late oogenesis [8,13,17] by two mechanisms: 1) the direct transport of cargo churns the cytoplasm [8,13] and 2) KHC-mediated microtubule sliding generates significant cytoplasmic flows [17]. Given that *Wolbachia’s* localization to the posterior pole begins at stage 9, and fast streaming begins in stage 10b, KHC-mediated transport must be direct. Consistent with this prediction, oocytes homozygous for hypomorphic KHC alleles capable of direct transport, but not streaming, exhibit normal *Wolbachia* distributions [5]. While streaming may not play a role in *Wolbachia* posterior transport, this KHC-mediated function may limit *Wolbachia’s* access to the motor protein. To investigate this question, we took advantage of a KHC mutant that specifically disrupts microtubule binding sites in the tail region of kinesin heavy chain [17].

Interestingly, elimination of microtubule binding in the *Khc[Kl*,*MutA]* mutant largely recapitulates the excess posterior *Wolbachia* localization phenotype of KHC overexpression (n = 17; Fig 2F and 2G–2J), with 35.7 +/- 19.5% of *Wolbachia* fluorescence at the posterior (p < = 1.09E-05) and a less extreme, 18.2 +/- 14.7% at the pole (p < = 9.36E-03). *Wolbachia* localization was analyzed in stocks homozygous for a *khc* null mutation bearing a transgene containing the *Khc[Kl*,*MutA]* null microtubule-sliding mutation or a wild-type copy of *khc* (*Khc[Kl*,*WT]*), which exhibited a wild-type *Wolbachia* localization pattern (n = 8; Fig 2E and 2G–2J and S1 Table). We propose that a likely explanation for these results is that by eliminating the microtubule domain on KHC, more KHC is available for *Wolbachia*-binding, thus increasing the effective concentration of the motor protein for bacterial transport.

### Overexpression of KHC does not significantly increase localization of key pole plasm component, Vasa

To assess how the localization of specific germline determinants responds to variations in KHC dosage, we imaged Vasa as a proxy for the pole plasm via antibody-labelling and a GFP-Vasa fusion protein construct (Fig 3 and S3G–S3I Fig). Vasa is a DEAD-box RNA-helicase protein involved in germ cell specification, oogenesis, transposon silencing, and posterior patterning [18]. In both antibody and GFP methods, a small amount of pole plasm in KHC-overexpressing oocytes appears to drift off of the posterior pole, whereas this is not observed in wild-type (Fig 3A–3E), and is consistent with previous reports [8,19]. Despite this qualitative phenotype, the amount of Vasa fluorescence at the posterior pole of KHC-overexpressing oocytes is not significantly different from wild-type (n = 9, p < = 0.7962; Fig 3I and S2 Table). Consistent with prior reports [17,19], deletion of the microtubule-sliding domain in *Khc[Kl*,*MutA]* had no effect on the total abundance of pole plasm, however it did appear to produce a more diffuse localization pattern (Fig 3F). Also as expected (see [19,20]), knockdown of KLC with RNAi disrupted Vasa localization, and increased its abundance relative to wild-type (Fig 3G; n = 8, p < = 9.32E-03, S2 Table). However, unlike *Wolbachia*, the heterozygous *Klc[1]* allele did not produce a significant increase in pole plasm abundance (Fig 3H).

### Knockdown of Milton increases posterior localization, but much less than KLC

To assess the nature of the interactions between *Wolbachia* and other KHC linker proteins, we tested knockdowns of Pat1 and Milton, which function in linking KLC [20] and mitochondria [21], respectively, to KHC. Oocytes expressing the insertion allele *Pat1*^*e02477*^ of the KLC-interacting protein Pat1 transported only slightly more *Wolbachia* to the posterior (n = 25; 33.29 +/- 26.84% (p < = 9.22E-04)) and posterior pole (n = 25, 26.29 +/- 23.65% (p < = 1.54E-03)) than wild-type (16.4 +/- 14.4% and 12.3 +/- 12.6% respectively), whereas the null allele *Pat1*^*robin*^ and the insertion allele *Pat1*^*EY15664*^ transported levels of *Wolbachia* to the posterior pole indistinguishable from wild-type (Fig 4 and S1 Table). These data indicate that *Wolbachia* does not compete with this protein to the same degree it does with Pat1’s binding partner KLC. While the relative % posterior and % pole values were not significant, *Pat1*^*robin*^ oocytes did exhibit significantly more total posterior and pole fluorescence than wild-type (n = 18, p < = 1.07E-04 and 3.50E-04, respectively). Knockdown of Milton by both of the tested RNAi constructs (see S1 Table) significantly increased *Wolbachia* transport, but Val 22 produced more consistent results, so we acquired more data for that genotype. On average, the Milton Val 22 RNAi oocytes exhibited 43.54 +/- 19.51% at the posterior and 34.44 +/- 16.81% at the pole (n = 23, p < 7.03E-09 and 2.91E-08, respectively), both significantly elevated relative to wild-type, consistent with a model whereby linker knockdown frees up KHC.

## Discussion

### *Wolbachia* transport to the posterior pole is independent of kinesin light chain

**Fig 5:**
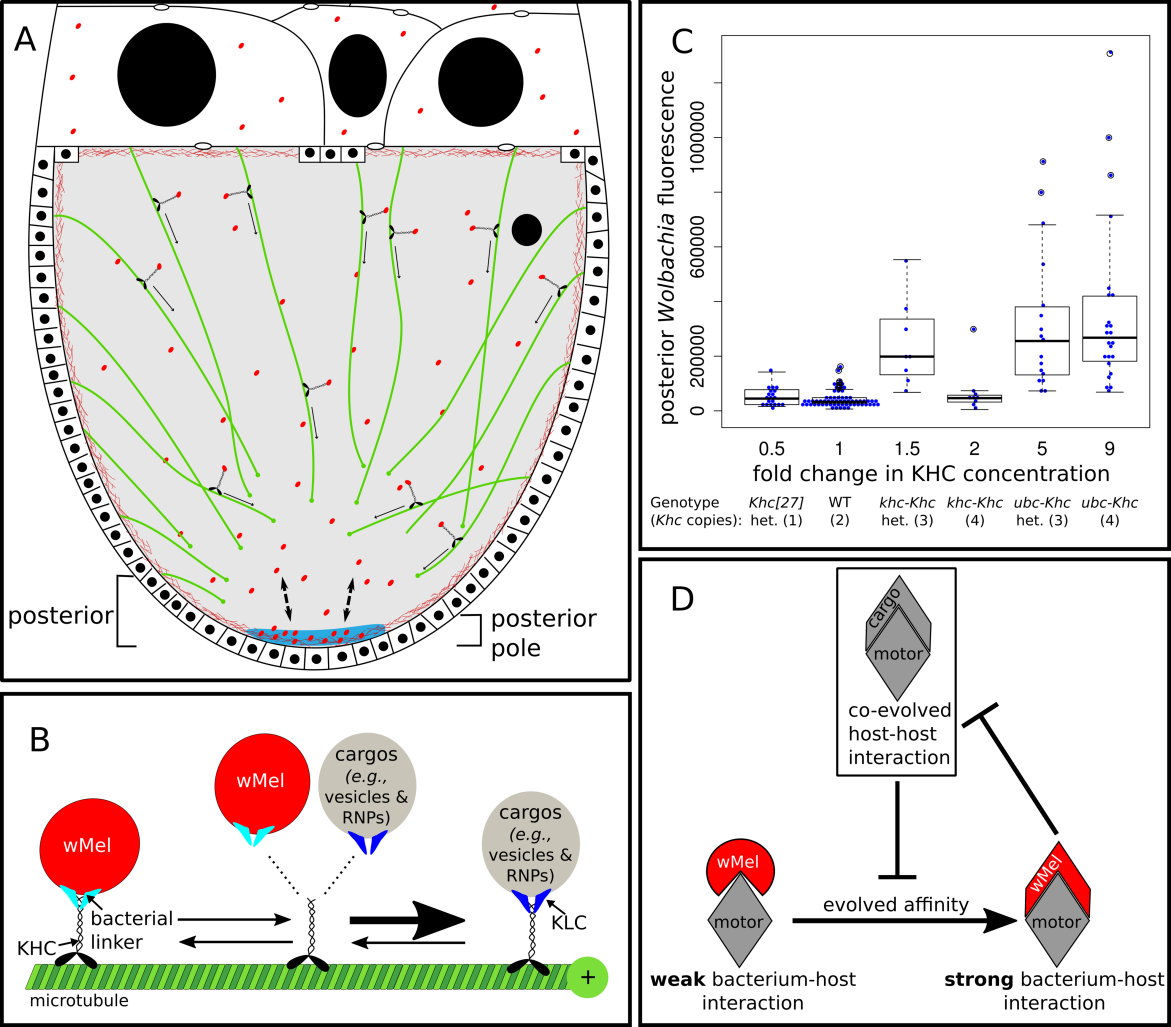
Model of *Wolbachia* competition for transport via host kinesin heavy chain (KHC). (A) Under normal conditions in stage 10a *D*. *melanogaster* oocytes, around 16% of *Wolbachia* in the oocyte are transported via KHC to the posterior where around 12% ultimately bind to the pole, coincident with the pole plasm (blue). When either more KHC is expressed or a strong competitor such as KLC is knocked down, more *Wolbachia* are able to bind KHC, resulting in more posterior transport. As the number of binding sites at the posterior pole have not increased, excess transport results in a cloud of unbound *Wolbachia* at the oocyte posterior (indicated by arrows between pole plasm (blue) and oocyte posterior). (B) At the molecular level, the wMel strain of *Wolbachia* competes with host KLC (blue) for binding to KHC using an unidentified linker protein of either host or bacterial (turquoise) origin for plus-end directed transport along microtubules (green). As represented by the width of the arrows, KHC has more affinity for binding KLC than *Wolbachia*. (C) Plot of posterior *Wolbachia* fluorescence versus concentration of KHC produced by various *Khc* alleles. KHC expression under the ubiquitin promoter was found to produce 4x the amount of KHC as expressed in wild-type [22]. (D). Evolutionary dynamic model for how *Wolbachia* is maintained as a poor competitor for host processes. After first entering a host lineage, a bacterial species should experience selective pressures to be a good competitor for host processes, ultimately resulting in evolved affinity for host factors. However, if too high of an affinity/effective a mimicry of host proteins occurs, then host fitness may suffer, which impacts the fitness of an obligately-vertically transmitted bacterium such as *Wolbachia*. Therefore, selection should limit the degree to which *Wolbachia* can evolve to compete for host processes.

Efficient maternal transmission from one generation to the next necessitates that *Wolbachia* are transported through the oocyte to the posteriorly localized host germplasm. Transport from the nurse cells to the anterior region of the oocyte relies on the minus-end motor dynein, and transport from the anterior to the posterior oocyte relies on plus-end directed Kinesin (Fig 5A [3,5]). To identify whether host KHC linker proteins are employed by *Wolbachia* to bind to KHC for posterior transport in the developing host oocyte, we screened mutants in candidate linker proteins for those that specifically disrupt *Wolbachia* posterior localization, finding a novel competitive interaction between host cargo and *Wolbachia* for KHC transport.

Previous studies demonstrated that *Wolbachia* posterior localization requires KHC [5], and KLC is required for much of KHC-dependent transport [8,9]. Thus, we examined *Wolbachia* localization in stage 10a *Drosophila* oocytes with severely reduced levels of KLC to test its necessity for transport of the bacteria. To our surprise, not only is *Wolbachia* able to localize to the posterior pole when KLC levels are reduced, but the level of *Wolbachia* localized at the pole increased. This result demonstrated that *Wolbachia* achieves its posterior localization through a mechanism independent of KLC. *Wolbachia* may bind KHC directly or through another linker protein [9], potentially of bacterial origin [11].

### Increasing KHC or reducing KLC levels results in increased *Wolbachia* localization at the posterior of the oocyte

An explanation for why knockdown of KLC levels results in an increase in posteriorly localized *Wolbachia* is that the bacteria compete with host cargos for access and association with KHC (depicted in Fig 5B). Knocking down KLC reduces the amount of KHC in the form of the heterotetramer Kinesin-1 and the number of KLC-dependent cargos binding KHC. This may increase the amount of KHC available to interact with *Wolbachia*. To test this idea, we overexpressed KHC and found a similar, yet more intense posterior localization phenotype than when KLC is knocked down. The severity of the phenotype was proportional to the degree of overexpression (see Fig 5C). Furthermore, selective knockdown of the microtubule-sliding function of KHC also recapitulates the overexpression phenotype. Together, these results indicate that *Wolbachia* competes with its host for use of KHC, and its transport is limited by the reduced concentration available for bacterial binding (Fig 5B). Given that KLC may play a role in regulating KHC [23], we note that this interpretation is consistent with an alternative mechanism in which KLC indirectly negatively regulates *Wolbachia* transport through signaling.

That KHC overexpression has little effect on the localization of the pole plasm component Vasa indicates that this competitive interaction, resulting in KHC-limitation for *Wolbachia*, is not a general property of pole plasm components and may be specific to *Wolbachia*. Indeed, KHC is not thought to be limiting for most host processes. For example, it was not found to be dosage sensitive in direct cellular transport [24], lipid droplet transport in early embryos [25], or cytoplasmic streaming [13,17]. While pole plasm localization, as quantified via Vasa-labeling, is clearly altered qualitatively by KHC overexpression (Fig 2B and 2H and [8]), our results show that the amount of pole plasm localized was not significantly different from wild-type. Furthermore, that KLC knockdown significantly alters pole plasm localization, whereas 9x KHC overexpression does not, also indicates that *Wolbachia* and transport of at least some pole plasm components are regulated differently. Given that in other insects, such as leafhoppers, posterior determinants associate and move with the endosymbiont, it is likely *Wolbachia* also tightly binds specific pole plasm components [12,26]. Identifying pole plasm components that are mislocalized with *Wolbachia* upon over-expression of KHC may provide a means of identifying putative host binding partners.

### *Wolbachia* may have evolved to weakly compete for association with host motor proteins

*Wolbachia* are present throughout oocyte development, and thus must avoid disrupting normal processes. During this period of maturation, the oocyte dramatically increases in volume and key determinants establishing the anterior/posterior and dorsal/ventral axes and germplasm are transported, positioned, and activated [27]. *Wolbachia* must replicate, migrate, and concentrate at the posterior pole without interfering with host oocyte development. Previous studies demonstrated that an over-abundance of *Wolbachia* in the oocyte disrupts axis formation [12], indicating that *Wolbachia* titer and localization must be strictly regulated. The studies here suggest that it is advantageous for *Wolbachia*, an obligate intracellular bacterium, to not disrupt key host cargo during the vertical transmission process, *i*.*e*., host development. Furthermore, while KHC-dependent transport is generally not dosage-dependent, there is likely some lower-limit on the amount needed for normal host functions, making *Wolbachia’s* relative affinity for it more important in these limiting situations.

Our finding that *Wolbachia* acts as a weak competitor for motor protein based transport contrasts with examples of viruses and other bacterial pathogens that outcompete host cargo for use of molecular motors [28,29]. It is likely that this difference is due to the fact that, despite being a manipulative, quasi-parasite, *Wolbachia’s* primary mode of transmission is vertical, so it is disadvantageous to be too effective at co-opting host proteins for its own use. Therefore, *Wolbachia* is likely to be strongly selected to compete poorly with host biology in order to avoid conflict and reduced host fitness (Fig 5D and [30]). Future work on other strains of *Wolbachia* and species of hosts that exhibit different localization patterns during oogenesis and have been co-evolving for different lengths of time will help determine how much competitive interactions at subcellular levels between *Wolbachia* and its hosts drive vertical transmission strategies.

## Materials and methods

### Fly strains

*wMel Wolbachia* were previously crossed into two *D*. *melanogaster* fly stocks, one carrying the markers and balancers *w[1]; Sp/Cyo*, *Sb/Tm6*, *Hu* and the other carrying the germline double driver: *P{GAL4-Nos*.*NGT}40*; *P{GAL4*::*VP16-Nos*.*UTR}MVD1*. These infected double balanced and ovary driver stocks were used to cross *wMel* into the null/hypomorphic mutants and RNAi TRiP lines to ensure that all *wMel* tested were of a similar genetic background. The *D*. *melanogaster* strains obtained from the Bloomington Drosophila Stock Center at the University of Indiana were: *P{lacW}Klc59A P{FRT(whs)}2A/TM6B*, *Tb+*, *y1 w67c23 P{EPgy2}Pat1[EY15664]*, *w[1118] PBac{RB}Pat1[e02477]*, y[1] v[1]; y1 w* Pat1[robin], *y1 v1; P{TRiP*.*HMC02365}attP2*, *P{TRiP*.*GL01515}attP2*. KHC-overexpression stocks *w[1]; Sp/CyO; P{w+ Khc+}3* and *w[1]; Sco/Cyo; P{w+ ub-Myc*::*Khc+}3*, which were obtained from Bill Saxton’s Lab at UCSC (from [15,16], respectively). The microtubule sliding mutant KHC and insertion control stocks, *w; Khc[KI*,*WT]/(CyO*, *Kr-Gal4*, *UAS-GFP*) and *w; Khc[KI*, *mutA]/(CyO*, *twist-Gal4(w+)*, *UAS-2XEGFP)*, were obtained from Vladimir Gelfand [17]. We obtained the Vas-GFP stock, w[*]; P{w[+mC] = vas.*EGFP*.*HA}2*, from the KYOTO Stock Center (DGRC) in the Kyoto Institute of Technology. All fly stocks and crosses were maintained at room temperature on white food (BDSC Cornmeal Food), as the sugar/protein composition of host food affects *Wolbachia* titer [31].

### Ovary fixation and PI staining

Newly enclosed flies were transferred to fresh white food and aged 3–5 days. As described previously [5], up to 10–15 flies from each cross were dissected, fixed, and stained with propidium iodide (PI) at a time. Briefly, ovaries were dissected, separating the ovarioles with pins, and fixed in 200 μl devitellinizing solution (2% paraformaldehyde and 0.5% v/v NP40 in 1x PBS) mixed with 600 μl heptane for 20 min at room temperature, with agitation. Then, oocytes were washed 5x in PBS-T (0.1% Triton X-100 in 1x PBS), and treated with RNAse A (10 mg/ml) overnight at room temperature. After washing six times in PBS-T, oocytes were incubated overnight in PI mounting media (20 μg/ml in 70% glycerol and 1x PBS), and mounted on glass slides. The *Wolbachia-*infected double balancer or *nos*-Gal4 driver stocks were used as wild-type controls. Experimental samples and control samples were processed simultaneously to minimize batch effects. Slides were imaged immediately or stored at -20°C degrees for no more than a week. While wild-type controls were processed alongside every experiment, oocyte localization variation within a run was no different from variation between runs, for wild-type and mutant genotypes, so these samples were pooled across processing runs to increase sample size and statistical power. Furthermore, no more than about five genotypes with 10 flies each could be dissected and processed at a time, making large, single-experiment sample sizes infeasible.

### Antibody staining

Ovaries were dissected and fixed as described for PI staining, except PBS-Tw (0.2% Tween 20 in 1x PBS) was used in the wash steps, and no more than five ovaries were prepared at a time to ensure proper Vas protein fixation. Following RNase A treatment overnight and washes, oocytes were blocked in 1% bovine serum albumin in PBS-Tw for one hr at room temperature. Then the ovaries were incubated in PBS-Tw containing anti-Vas antibody (Developmental Studies Hybridoma Bank) at a 1:200 dilution overnight at 4°C. The next day, following four washes in PBS-Tw, oocytes were incubated in secondary antibody (Alexa 633 conjugated goat anti rat, Invitrogen) at a 1:500 dilution in PBS-Tw overnight at 4°C. Following four washes in PBS-Tw, ovaries were incubated overnight in PI mounting media and then mounted on glass slides, as described above.

### Embryo collection, fixation, and staining

KHC homozygous virgins (males and females) were collected for 1–3 days before being crossed. Flies laid embryos for 3 hrs at room temperature on grape juice containing agar plates topped with white food. The collection period lasted 2–4 days. Embryos were collected and dechorinated in a 50% bleach solution for 1–2 min, and rinsed in DI water for 1 min. Embryos were then transferred into a 1:1 volume ratio of heptane and 37% formaldehyde for 5 min. The formaldehyde was removed and embryos were placed in a 1:1 solution of heptane and methanol. Heptane was removed and embryos were stored in methanol at 4 degrees. The embryos were rehydrated in PBTA (phosphate-buffered saline [PBS] + 0.1% Triton X-100 + 0.05% sodium azide) before staining. The embryos were then incubated in RNAse at 37°C for 2 hrs. After 4 washes of PBTA, the embryos were mounted in mounting media containing propidium iodide (PI) and viewed with confocal microscopy. Slides were stored at -20°C degrees.

### MitoTracker staining

Following [32], oocytes of 5-day old females were dissected in cold 1x PBS and ovarioles were separated. Oocytes were incubated on slides in a mixture of 1:100 Syto 11 (5 mM; Invitrogen) and 1:1000 MitoTracker Red (Molecular Probes, M-7512) in 1x PBS on ice for 20 min in the dark. Coverslips were applied, slides were stored on ice, and oocytes were imaged on an SP5 confocal microscope before 145 total minutes had elapsed.

### Confocal imaging and analysis

Oocytes were imaged on a Leica SP5 confocal microscope with a 63x objective. Optical sections were taken at the Nyquist value for the objective, every 0.38 μm, at a magnification of 1.5x. Propidium iodide was excited with the 514 and 543 nm lasers, and emission from 550 to 680 nm was collected. GFP was imaged with the 488 nm laser, and emission from 488 to 540 nm was collected. Alexa 633 was imaged with the 633 laser, and emission from 606 to 700 nm was collected.

### Image analysis

Approximately one μm thick sections were 3D reconstructed from three sections near the middle plane of the oocytes in ImageJ. For fluorescence quantification, the selection tool in ImageJ was used to isolate the oocyte and then the brightness/contrast tool was used to increase the threshold on the image to the point that only white puncta corresponding to *Wolbachia* staining were retained, and all background noise was rendered black (see S1 Fig for a visual explanation). Fluorescence intensity was measured by setting ImageJ to measure: AREA, INTEGRATED DENSITY and MEAN GRAY VALUE, and measuring: three sample background selections, the whole oocyte, the posterior region, and the posterior pole. Then the corrected total cell fluorescence (CTCF) was calculated for each region of the oocyte as follows:

CTCF = Raw Integrated Density–Area of selected cell X Mean fluorescence of background readings

### Plotting and statistical analysis

The resulting fluorescent intensities and relative proportions of fluorescence intensity were plotted, analyzed, and statistics were calculated in R. Total and relative fluorescence intensities between oocyte genotypes were compared with the nonparametric Wilcoxon Rank Sum test. See Figs 1–4, Fig 5C, S2 Fig, S1 Table, S2 Table

## Supporting information

S1 Fig Oocyte *Wolbachia* quantification. Comparing a propidium iodide (PI)-stained (A) uninfected *D*. *melanogaster* oocyte with a (B) *Wolbachia*-infected one, the fluorescence due to the bacteria in the cytoplasm of the oocyte is clear to see. (C-E) To quantify oocyte fluorescence, the cytoplasm of the infected oocytes was selected in ImageJ, image adjusted (brightness and contrast) so as to only see fluorescence due to *Wolbachia*, and measurements taken from the following regions: (A) total oocyte, (D) posterior oocyte, and (E) posterior pole of oocyte.
(TIF)

S2 Fig Overexpression of kinesin heavy chain (KHC) lowers *Wolbachia* abundance in nurse cells of 10a oocytes. (A-D) Confocal micrographs of *D*. *melanogaster* nurse cells stained with propidium iodide (PI) showing representative examples of (A) wild-type (WT) localization of *Wolbachia*, (B) localization under KLC RNAi knockdown and (C) KHC overexpression. (D) Quantification of PI fluorescence due to *Wolbachia*. Plots are colored according to their genotype label colors in A-C. Violin plot of the total fluorescence due to *Wolbachia* in the nurse cells (nuclei removed) for each of the genotypes listed on the x-axes. Genotypes that contained significantly different posterior abundances than WT in Wilcoxon rank sum tests: * p < = 0.01. Scale bars = 25 μm.
(TIF)

S3 Fig Impacts of excess *Wolbachia* localization on subsequent stages of development and overall transmission. Confocal micrographs of *D*. *melanogaster* (A,B) oocytes and (C-F) embryos stained with propidium iodide (PI) showing representative examples of (A,C,E) wild-type (WT) *Wolbachia* localization and (B,D,F) the impact of KHC-overexpression on localization. (A,B) Excess *Wolbachia* clump and drift in the cytoplasm in *ubc-Khc++* oocytes after cytoplasmic streaming begins in stage 10b (arrowhead in B). (C-D) Posterior of cycle 9 embryos and (E-F) pole cells of cycle 11 embryos. (G,H) Continued from Fig 3, overexpression of kinesin heavy chain (KHC) does not significantly increase localization of the pole plasm component Vasa (Vas) in stage 10a oocytes. Confocal micrographs of fixed *D*. *melanogaster* oocytes with Vasa protein localized by immunolabeling (green) and stained with propidium iodide (red). Oocytes are infected with wMel *Wolbachia*. Magnified views of (G’,H’) pole region in (G,H) and (G”,H”) pole region in for Vasa-labelling only, respectively. Quantification of Vas fluorescence signal at the oocyte posterior pole in G,H is shown in Fig 3 and S2 Table. (A-F) Scale bars = 50 μm. (G,H) Scale bars = 25 μm.
(TIF)

S4 Fig Oocyte fluorescence data presented for all genotypes, replotted from Figs 1, 2 and 4, for direct comparison among genes and genotypes. A) The total fluorescence in each oocyte plotted against the quantity of that fluorescence localized at the oocyte posterior or B) posterior pole, along the cortex. Colors as in Figs 1, 2 and 4, but plotted at 50% transparency to show overlapping data.
(TIF)

S1 Table *Wolbachia* oocyte quantification values, with p-values < = 0.01 in bold. (PDF)

S2 Table Vasa oocyte quantifications values with p-values < = 0.01 in bold. (PDF)
