# Correction: *Wolbachia* and host germline components compete for kinesin-mediated transport to the posterior pole of the *Drosophila* oocyte

**DOI:** 10.1371/journal.ppat.1007557

**Published:** 2019-01-30

**Authors:** Shelbi L. Russell, Nassim Lemseffer, William T. Sullivan

Dr. Pamela White should be included in the author byline. She should be listed as the third author, and her affiliation is 1: Department of Molecular, Cell and Developmental Biology, University of California, Santa Cruz, Santa Cruz, CA. The contributions of this author are as follows: Conceptualization, Investigation, Supervision.

The correct citation is: Russell SL, Lemseffer N, White PM, Sullivan WT (2018) *Wolbachia* and host germline components compete for kinesin-mediated transport to the posterior pole of the *Drosophila* oocyte. PLoS Pathog 14(8): e1007216. https://doi.org/10.1371/journal.ppat.1007216
