# Supplementary figures and images for: Wolbachia and host germline components compete for kinesin-mediated transport to the posterior pole of the Drosophila oocyte
Source: PLoS Pathog. 2018 Aug 15;14(8):e1007216. doi: 10.1371/journal.ppat.1007216 (PMC6110520; doi:10.1371/journal.ppat.1007216)

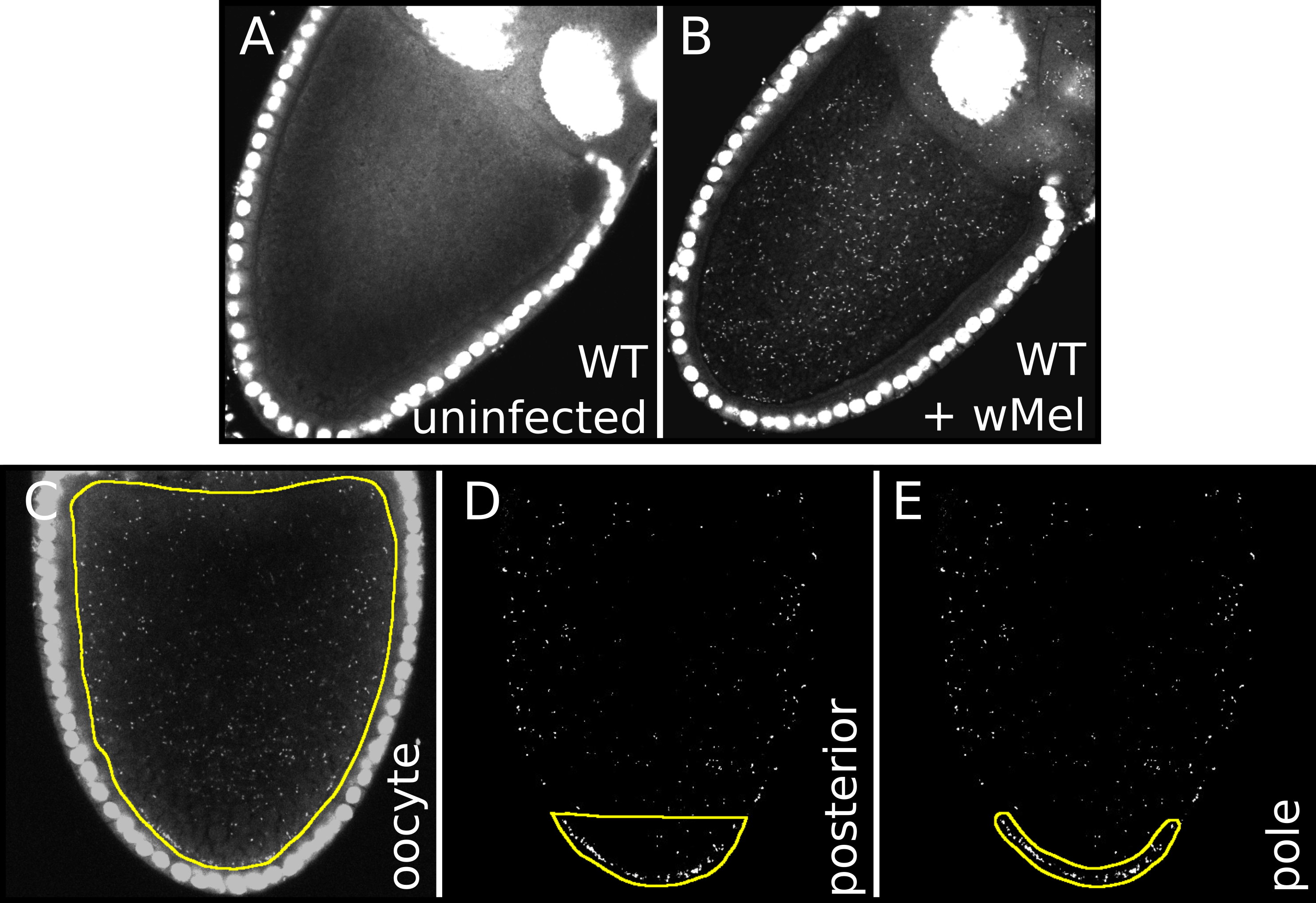

Supplement: S1 Fig — Comparing a propidium iodide (PI)-stained (A) uninfected D. melanogaster oocyte with a (B) Wolbachia-infected one, the fluorescence due to the bacteria in the cytoplasm of the oocyte is clear to see. (C-E) To quantify oocyte fluorescence, the cytoplasm of the infected oocytes was selected in ImageJ, image adjusted (brightness and contrast) so as to only see fluorescence due to Wolbachia, and measurements taken from the following regions: (A) total oocyte, (D) posterior oocyte, and (E) posterior pole of oocyte. (TIF) [file ppat.1007216.s001.tif]

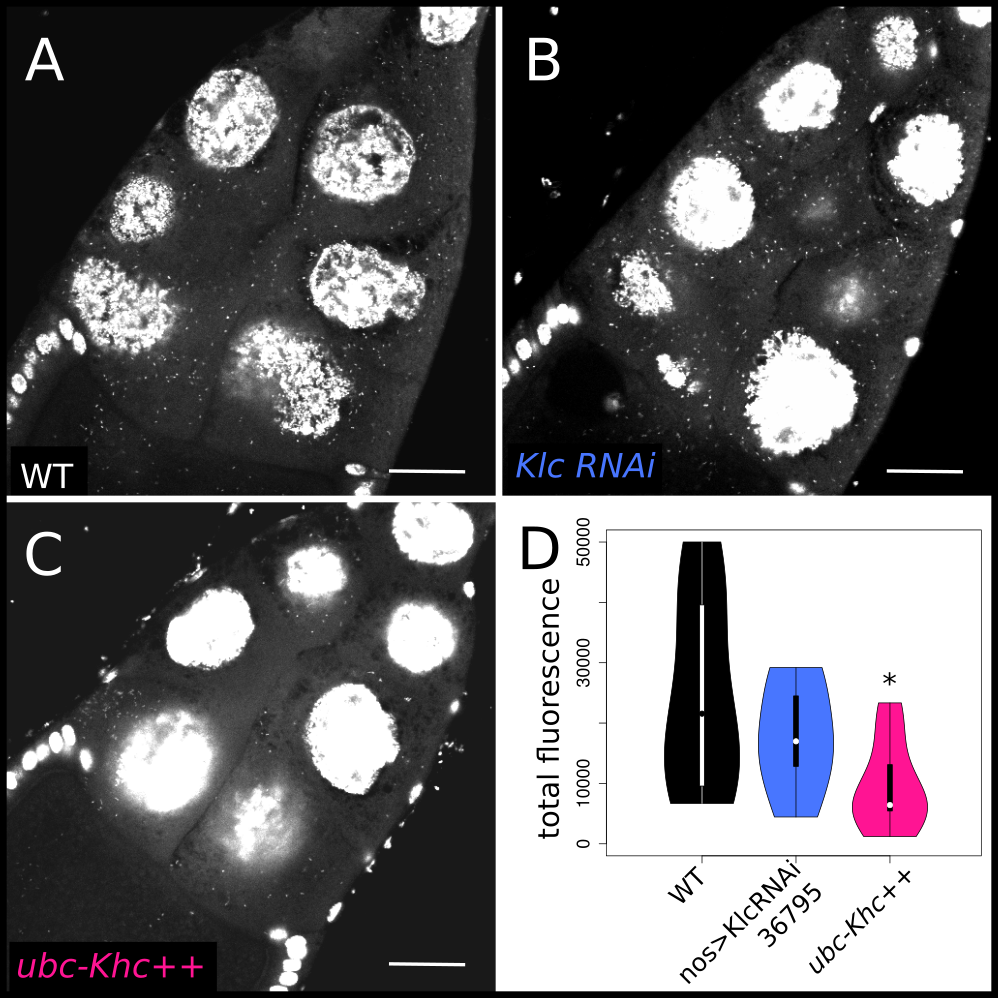

Supplement: S2 Fig — (A-D) Confocal micrographs of D. melanogaster nurse cells stained with propidium iodide (PI) showing representative examples of (A) wild-type (WT) localization of Wolbachia, (B) localization under KLC RNAi knockdown and (C) KHC overexpression. (D) Quantification of PI fluorescence due to Wolbachia. Plots are colored according to their genotype label colors in A-C. Violin plot of the total fluorescence due to Wolbachia in the nurse cells (nuclei removed) for each of the genotypes listed on the x-axes. Genotypes that contained significantly different posterior abundances than WT in Wilcoxon rank sum tests: * p < = 0.01. Scale bars = 25 μm. (TIF) [file ppat.1007216.s002.tif]

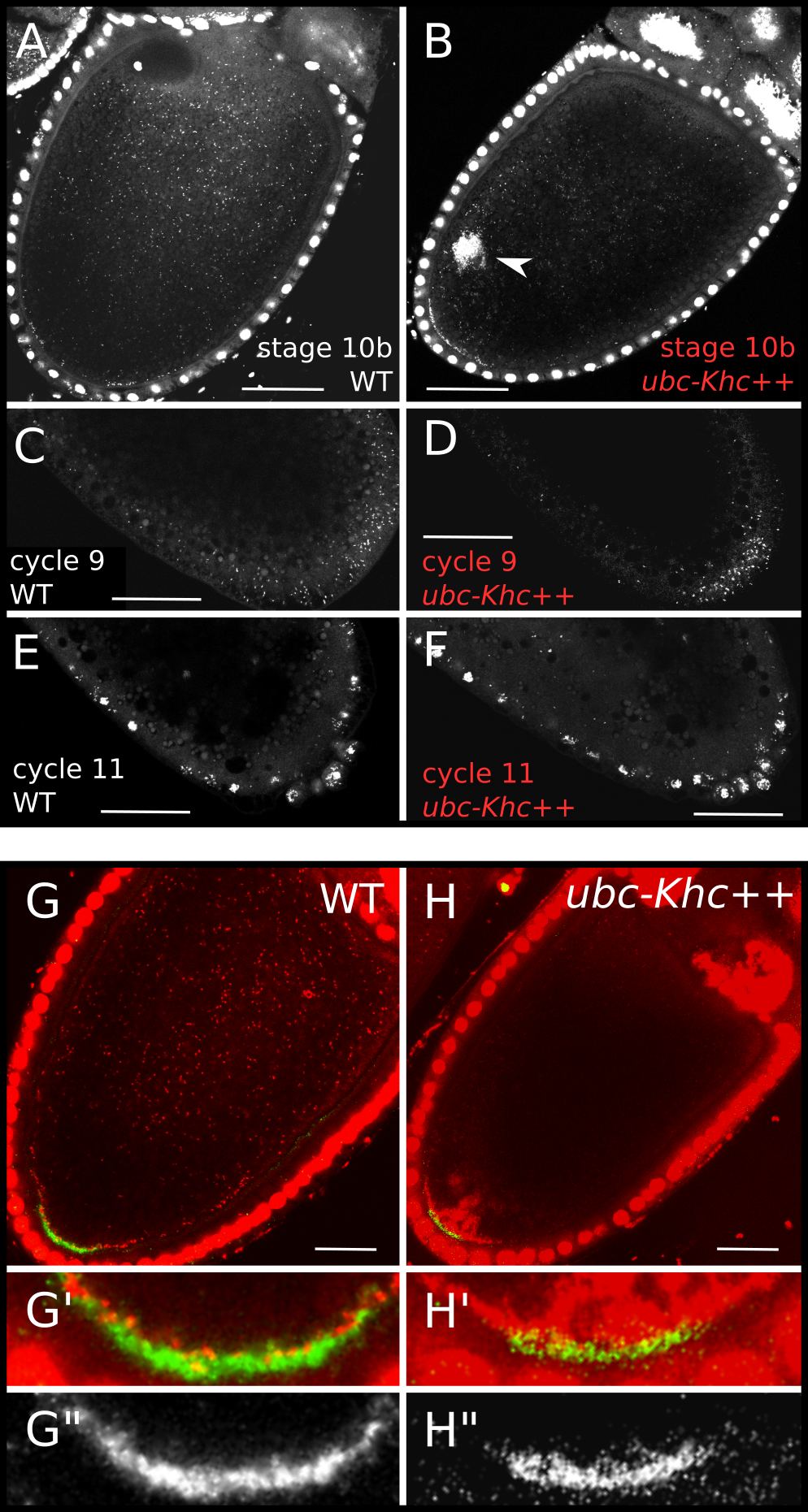

Supplement: S3 Fig — Confocal micrographs of D. melanogaster (A,B) oocytes and (C-F) embryos stained with propidium iodide (PI) showing representative examples of (A,C,E) wild-type (WT) Wolbachia localization and (B,D,F) the impact of KHC-overexpression on localization. (A,B) Excess Wolbachia clump and drift in the cytoplasm in ubc-Khc++ oocytes after cytoplasmic streaming begins in stage 10b (arrowhead in B). (C-D) Posterior of cycle 9 embryos and (E-F) pole cells of cycle 11 embryos. (G,H) Continued from Fig 3, overexpression of kinesin heavy chain (KHC) does not significantly increase localization of the pole plasm component Vasa (Vas) in stage 10a oocytes. Confocal micrographs of fixed D. melanogaster oocytes with Vasa protein localized by immunolabeling (green) and stained with propidium iodide (red). Oocytes are infected with wMel Wolbachia. Magnified views of (G’,H’) pole region in (G,H) and (G”,H”) pole region in for Vasa-labelling only, respectively. Quantification of Vas fluorescence signal at the oocyte posterior pole in G,H is shown in Fig 3 and S2 Table. (A-F) Scale bars = 50 μm. (G,H) Scale bars = 25 μm. (TIF) [file ppat.1007216.s003.tif]

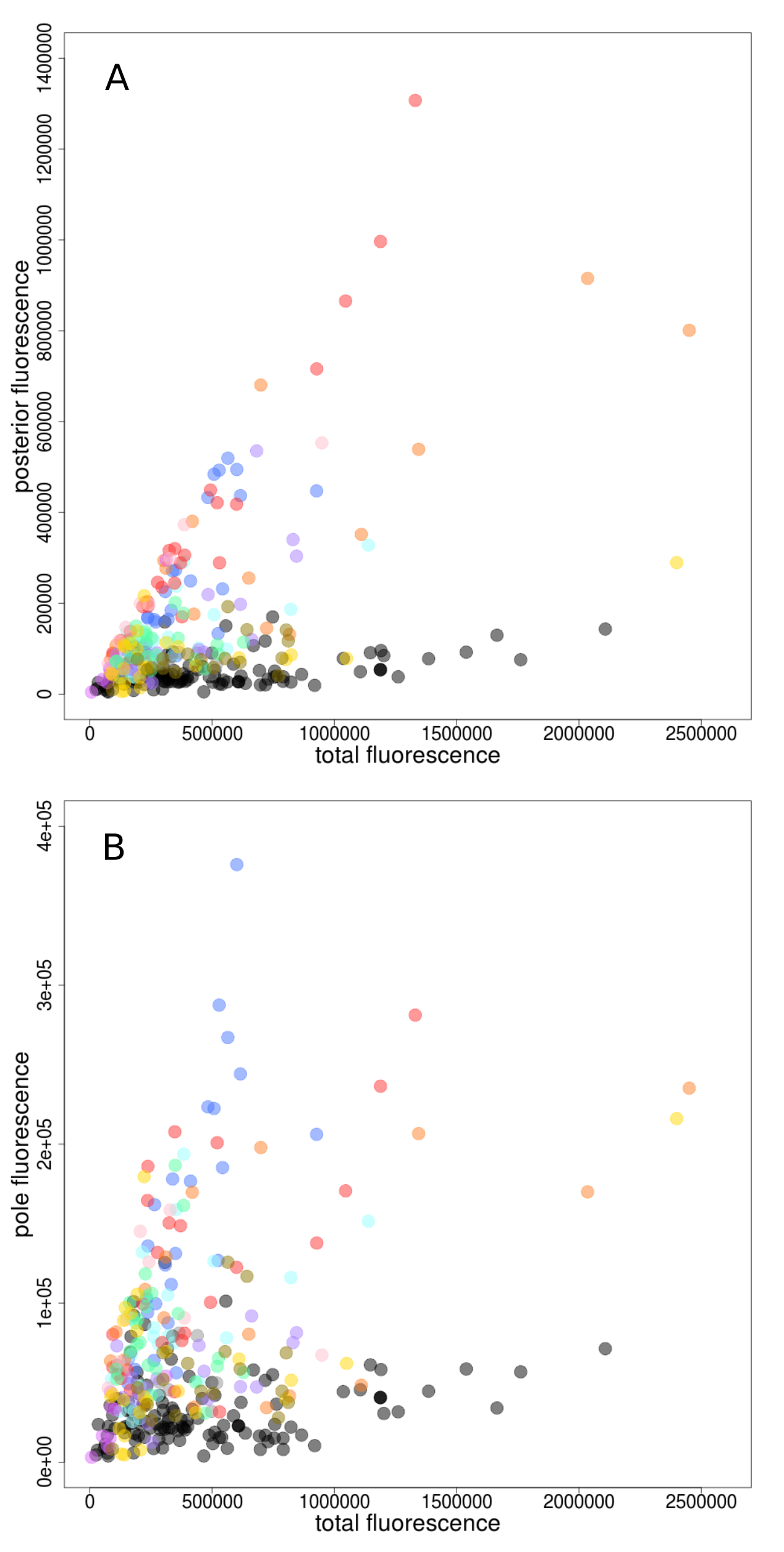

Supplement: S4 Fig — A) The total fluorescence in each oocyte plotted against the quantity of that fluorescence localized at the oocyte posterior or B) posterior pole, along the cortex. Colors as in Figs 1, 2 and 4, but plotted at 50% transparency to show overlapping data. (TIF) [file ppat.1007216.s004.tif]
