# Supplementary material for: Wolbachia and host germline components compete for kinesin-mediated transport to the posterior pole of the Drosophila oocyte
Source: PLoS Pathog. 2018 Aug 15;14(8):e1007216. doi: 10.1371/journal.ppat.1007216 (PMC6110520; doi:10.1371/journal.ppat.1007216)
Supplement: S1 Table — (PDF) [file ppat.1007216.s005.pdf]

gene	genotype	name in text	stock	N	average total fluorescence	standard deviation	Wilcoxon rank sum p-value	average posterior fluorescence	standard deviation	Wilcoxon rank sum p-value	average pole fluorescence	standard deviation	Wilcoxon rank sum p-value	average % posterior fluorescence	standard deviation	Wilcoxon rank sum p-value	average % pole fluorescence	standard deviation	Wilcoxon rank sum p-value
NA	w; Sp/CyO; Sb/Hu or nos-Gal4; nos-Gal4	WT	double balancer or nos-Gal4 driver	125	4.64E+05	3.93E+05	NA	4.73E+04	3.22E+04	NA	3.22E+04	2.20E+04	NA	16.37	14.38	NA	12.29	12.59	NA
klc	P{TRiP.GL00535} attP40	Klc RNAi (Val22)	Bloomington 36795	26	3.78E+05	1.86E+05	9.63E-01	2.43E+05	1.55E+05	**1.46E-13**	1.50E+05	8.35E+04	**5.33E-14**	62.36	20.48	**1.70E-13**	40.30	12.28	**3.48E-12**
klc	w*; P{lacW}Klc[59A] P {FRT(whs)}2A/TM6B, Tb+	Klc[1]/WT (aka Klc [59A]) heterozygous null	Bloomington 31996	27	3.13E+05	2.29E+05	7.22E-02	1.13E+05	7.47E+04	**1.56E-08**	7.97E+04	4.37E+04	**2.47E-09**	40.02	19.40	**6.91E-10**	30.09	15.79	**7.36E-09**
khc	w; Sp/CyO; P{w+ Khc+}3	*khc-Khc+* overexpression	from Saxton Lab (Brendza et al. 1999)	9	9.79E+04	8.50E+04	**9.53E-05**	7.05E+04	8.65E+04	4.88E-01	2.44E+04	1.77E+04	2.74E-01	63.64	15.94	**2.21E-06**	28.88	14.36	**5.72E-04**
		khc-Khc+/WT heterozygote overexpression		8	3.07E+05	2.80E+05	2.13E-01	2.44E+05	1.59E+05	**1.01E-05**	9.84E+04	4.07E+04	**2.29E-05**	89.04	13.75	**2.50E-06**	47.16	21.28	**1.24E-04**
khc	w; Sco/Cyo; P{w+ ub-Myc::Khc+}3	*ubc-Khc++ overexpression*	from Saxton Lab (Brendza et al. 2000)	24	4.45E+05	3.44E+05	9.36E-01	3.62E+05	3.10E+05	**1.34E-13**	1.23E+05	6.71E+04	**8.53E-12**	80.55	14.67	**2.45E-14**	36.68	21.84	**1.04E-08**
		ubc-Khc++/WT heterozygote overexpression		17	7.10E+05	6.80E+05	1.73E-01	3.25E+05	2.60E+05	**3.43E-10**	1.06E+05	6.68E+04	**8.90E-08**	60.58	31.06	**1.80E-08**	27.59	23.48	**2.03E-03**
khc	w; Khc[KI,WT]/(CyO, Kr-Gal4, UAS-GFP)	*Khc[KI,WT]* wild type allele insertion	from Gelfand Lab (Lu et al. 2016)	8	3.43E+05	1.42E+05	8.02E-01	6.69E+04	1.89E+04	**9.13E-03**	5.31E+04	1.90E+04	**3.96E-03**	23.26	12.41	4.05E-02	19.05	12.99	2.99E-02
khc	w; Khc[KI, mutA]/ (CyO, twist-Gal4(w+), UAS-2XEGFP)	*Khc[KI,MutA]* Insertion of Khc lacking microtubule-binding residues	from Gelfand Lab (Lu et al. 2016)	17	4.02E+05	2.47E+05	9.15E-01	1.45E+05	1.36E+05	**2.62E-05**	5.29E+04	2.07E+04	**1.23E-04**	35.65	19.52	**1.09E-05**	18.16	14.68	**9.36E-03**
khc	w; neoFRT42B Khc [27]/CyO	*Khc[27]*/WT heterozygous null	Sullivan Lab	20	3.61E+05	2.07E+05	7.12E-01	5.11E+04	3.24E+04	7.20E-01	3.20E+04	2.24E+04	8.25E-01	16.43	9.57	3.54E-01	11.45	8.60	7.27E-01
Pat1	y1 w67c23 P{EPgy2} Pat1[EY15664]	*Pat1[EY15664]* insertion allele	Bloomington 21151	3	4.15E+04	1.46E+04	**4.58E-03**	2.33E+04	1.76E+04	1.71E-01	1.72E+04	1.17E+04	2.64E-01	49.40	29.46	3.62E-02	37.90	21.93	2.33E-02
Pat1	w1118 PBac{RB}Pat1 [e02477]	*Pat1[e02477]* insertion allele	Bloomington 18059	25	3.83E+05	4.91E+05	4.98E-02	8.17E+04	6.40E+04	**2.70E-03**	6.07E+04	5.05E+04	**1.34E-03**	33.29	26.84	**9.22E-04**	26.29	23.65	**1.54E-03**
Pat1	y1 w* Pat1[robin]	*Pat1[robin]* null allele	Bloomington 67697	18	4.79E+05	2.28E+05	1.98E-01	8.14E+04	4.30E+04	**1.07E-04**	5.38E+04	2.97E+04	**3.50E-04**	18.58	8.70	4.37E-02	12.60	6.34	8.89E-02
milton	y1 v1; P{TRiP. HMC02365}attP2	Milton RNAi (Val20)	Bloomington 44477	16	3.44E+05	3.25E+05	1.02E-01	7.62E+04	3.36E+04	**7.33E-04**	5.27E+04	2.42E+04	**4.32E-04**	40.46	23.82	**4.65E-05**	32.75	23.61	**4.59E-04**
milton	P{TRiP.GL01515} attP2	Milton RNAi (Val22)	Bloomington 43173	23	2.91E+05	1.42E+05	1.21E-01	1.06E+05	3.71E+04	**1.19E-09**	8.27E+04	3.68E+04	**2.14E-10**	43.54	19.51	**7.03E-09**	34.44	16.81	**2.91E-08**

S1 Table. Wolbachia oocyte quantification values, with p-values <= 0.01 in bold.
