# Supplementary material for: Wolbachia and host germline components compete for kinesin-mediated transport to the posterior pole of the Drosophila oocyte
Source: PLoS Pathog. 2018 Aug 15;14(8):e1007216. doi: 10.1371/journal.ppat.1007216 (PMC6110520; doi:10.1371/journal.ppat.1007216)
Supplement: S2 Table — (PDF) [file ppat.1007216.s006.pdf]

gene	genotype	name in text	stock	status	Vas-detection method	N	average pole fluorescence	standard deviation	Wilcoxon rank sum p-value
NA	w; Sp/CyO; Sb/Hu or nos-Gal4; nos-Gal4	WT	double balancer or nos-Gal4 driver	uninfected	antibody	9	5.48E+04	1.90E+04	2.99E-01
				wMel infected	antibody	7	7.87E+04	4.08E+04	NA
				wMel infected	GFP-transgene	11	5.16E+04	2.94E+04	1.51E-01
klc	P{TRiP.GL00535}attP40	Klc RNAi (Val22)	Bloomington 36795	wMel infected	antibody	8	1.59E+05	5.77E+04	**9.32E-03**
klc	w*; P{lacW}Klc59A P{FRT (whs)}2A/TM6B, Tb+	*Klc[1]*/WT (aka *Klc[59A])* heterozygous null allele	Bloomington 31996	wMel infected	antibody	6	1.18E+05	5.58E+04	2.34E-01
khc	w; Sco/Cyo; P{w+ ub-Myc:: Khc+}3	*ubc-Khc++* overexpression	from Saxton Lab (Brendza et al. 2000)	uninfected	antibody	9	6.47E+04	3.40E+04	7.96E-01
				wMel infected	antibody	2	5.91E+04	1.35E+04	5.00E-01
				wMel infected	GFP-transgene	2	3.68E+04	4.75E+03	4.10E-01
khc	Khc[Kl, MutA]	*Khc[Kl,MutA] Insertion of Khc lacking microtubule-binding residues*	from Gelfand Lab (Lu et al. 2016)	wMel infected	antibody	5	7.38E+04	3.14E+04	1.00E+00

S2 Table. Vasa oocyte quantifications values with p-values <= 0.01 in bold.
